# Editorial

**Published:** 1917-01

**Authors:** 


### EDITORIAL

The Atlanta Journal-Record of Medicine has its Business Office at 23 West Alabama Street where all business communications should be sent.

Remittances are payable to The Journal-Record of Medicine.

Reprints of Original Articles will be furnished at cost price. Requests for them had best be made on the Manuscript.

Upon request, we will present, postpaid, to each contributor of an original article, twenty copies of The Journal-Record of Medicine containing such article.

---

### ADDRESS OF DR. JAMES B. BAIRD AT COMPLIMENTARY DINNER TO DR. J. G. EARNEST.

Mr. Chairman and Gentlemen:

Your generous partiality has conferred upon me the privilege of representing you and of speaking for you, in extending tonight cordial greeting to our distinguished friend—our honored guest. In performing the pleasing task assigned me, I shall ask your indulgence and his forbearance, while I offer a few pertinent remarks appropriate to this interesting occasion—his fiftieth medical birthday.

The beloved bard of Scotland, after his own fascinating style, once exclaimed:

> "Oh wad some power the gifts gie us
> To see oursels as ithers see us,
> It would 'fra mony a blunder frae us,
> And foolish notion."

It certainly is inconsistent with my desire to cause our good friend any alarm by proposing to hold before him the mirror of popular estimation. The sole purpose in showing him to himself is to present for his inspection an image true to nature, without either detraction or embellishment, and I am sure it will not embarrass him. But, in a recent number of a literary magazine the question was editorially asked,

"What is the matter with the medical profession?" Many answers from well known persons—mostly authors of note—were published in the form of a symposium. The preponderance of evidence—as our lawyer friends would say—especially in the case of expert medical testimony—was in favor of the doctor. But the favorable opinion expressed was, by no means, unanimous. There was much criticism, more or less severe, and some censure. The unfavorable comments were, for the most part, directed toward unworthy members. Which fact strongly emphasizes the individual obligation of all the members of a profession to uphold and to maintain its standard by adequate preparedness and by scrupulous personal conduct.

Those of us who can look back for several decades, must realize that the influence of our profession has sadly waned within late years, and I do not hesitate to assert that this unfortunate fact is the fault of the profession itself. This is not the time or the place to discuss this unhappy condition. My object in referring to it here, is to lay stress upon the quality and the standing of the unit as affecting and determining the value of the aggregate. If the units are right, the aggregate will be right. If some of the units are wrong, the aggregate must of necessity suffer.

Far be it from me, Mr. Chairman, to detract from the dignity of this occasion by indulgence in mere empty phrases or by bestowing fulsome praise, and the friends of Dr. Earnest who know him best will readily acquit our tribute of all excess.

His long professional life—as the lives of mortals go—has been characterized by unfaltering fidelity, by patience, by courtesy and by an intelligent conception of his relations to his fellow-man. His career has been marked by unvarying adherence to duty. He has not yielded to the craze for novelties, and has not been guilty of the unreasoning or the mercenary use of them. Experience has taught him what it teaches every intelligent and thoughtful man, that all that is new is not good, and all that is good is not new. No taint of commercialism, no blight of avarice, no greed for gain attaches to him. Neither has his soul been distorted or shriveled by jealousy. Illustrating the noblest type of the practical physician, and as a worthy unit in the ranks of the profession, his example invites emulation. He has acted well his part. He has been true to himself.

Trials, sorrows and disappointments—inevitable experiences—have overtaken him, but they have not marred his high character, altered his fixed purpose or changed his onward course. Nay, rather have they served to stimulate and to strengthen his resolute determination to meet every obligation and faithfully to discharge every trust. So, as dessert is better than achievement, regardless of his successes, nothing of failure may be attributed to him.

Therefore, hônored sir, in behalf of this assembled company and of others, perhaps, not present, I ask you to accept this loving cup as a feeble expression of our confidence, our regard and our friendship—as a token of our loyalty to virtue, of our admiration for your qualities and of our affection for you, with the fervent wish that a benignant Providence may long continue to shed upon your pathway a kindly light, as a guide, an inspiration and as an incentive to steadfast allegience to the cause of well-doing, until finally, in his own good time, and in the great hereafter, where it is written of you "He loved his fellow-men," gathered among the faithful and the deserving, you shall receive, for a well-spent life, your eternal reward.

